# Trained Immunity or Tolerance: Opposing Functional Programs Induced in Human Monocytes after Engagement of Various Pattern Recognition Receptors

**DOI:** 10.1128/CVI.00688-13

**Published:** 2014-04

**Authors:** Daniela C. Ifrim, Jessica Quintin, Leo A. B. Joosten, Cor Jacobs, Trees Jansen, Liesbeth Jacobs, Neil A. R. Gow, David L. Williams, Jos W. M. van der Meer, Mihai G. Netea

**Affiliations:** a Radboud University Medical Center, Department of Internal Medicine, Division of Experimental Internal Medicine, Nijmegen, The Netherlands; b Aberdeen Fungal Group, School of Medical Sciences, Institute of Medical Sciences, University of Aberdeen, Aberdeen, United Kingdom; c Department of Surgery, James H. Quillen College of Medicine, East Tennessee State University, Johnson City, Tennessee, USA

## Abstract

Upon priming with Candida albicans or with the fungal cell wall component β-glucan, monocytes respond with an increased cytokine production upon restimulation, a phenomenon termed “trained immunity.” In contrast, the prestimulation of monocytes with lipopolysaccharide has long been known to induce tolerance. Because the vast majority of commensal microorganisms belong to bacterial or viral phyla, we sought to systematically investigate the functional reprogramming of monocytes induced by the stimulation of pattern recognition receptors (PRRs) with various bacterial or viral ligands. Monocytes were functionally programmed for either enhanced (training) or decreased (tolerance) cytokine production, depending on the type and concentration of ligand they encountered. The functional reprogramming of monocytes was also associated with cell shape, granulocity, and cell surface marker modifications. The training effect required p38- and Jun N-terminal protein kinase (JNK)-mediated mitogen-activated protein kinase (MAPK) signaling, with specific signaling patterns directing the functional fate of the cell. The long-term effects on the function of monocytes were mediated by epigenetic events, with both histone methylation and acetylation inhibitors blocking the training effects. In conclusion, our experiments identify the ability of monocytes to acquire adaptive characteristics after prior activation with a wide variety of ligands. Trained immunity and tolerance are two distinct and opposing functional programs induced by the specific microbial ligands engaging the monocytes.

## INTRODUCTION

More than 3 decades ago, a number of studies reported enhanced immune responses upon reinfection in diverse invertebrate taxa that do not possess adaptive immunity, such as cockroaches (1–3), shrimp (4, 5), and mealworm beetles (6). Interestingly, invertebrates also have proven to be able to mount enhanced secondary immune responses (7) and to transmit protection to their offspring (8). While this protection is nonspecific, several studies demonstrated that these effects enable discrimination between different classes of pathogens. Recently, Witteveldt et al. (9) showed that such immune memory can be used in the vaccination of invertebrates. Whereas vertebrates use somatic rearrangement of immunological receptors to induce adaptive immune responses, one mechanism employed by the host defenses of invertebrates to confer adaptation to infection is alternative splicing of pattern recognition genes, such as Down syndrome cell adhesion molecule, which generates a highly diverse set of >31,000 potential alternative splice forms (10, 11). These two molecular processes have the same consequence: they create a receptor repertoire that is sufficiently diverse for discriminating between the broad varieties of different antigens.

The function of prototypic mammalian innate immune cells, such as NK cells, can also be enforced, leading to protection against reinfection with viral pathogens (12–15). Similarly, monocytes and/or macrophages exhibit memory characteristics that mediate protective effects after a second encounter with a pathogen (16, 17). We have termed this phenomenon “trained immunity,” defined as enhanced nonspecific innate immune protection that is suggested to be mediated by epigenetic mechanisms (18). In contrast to trained immunity, engagement of the Toll-like receptor 4 (TLR4) by lipopolysaccharide (LPS) has been known for several decades to inhibit the cell function in a process called LPS-induced tolerance (19, 20). LPS tolerance is an active process involving epigenetic remodeling (21), and this process has been suggested to be the basic mechanism responsible for the immunoparalysis that occurs after Gram-negative sepsis (22).

Despite the importance of both LPS tolerance (19, 20) and trained immunity (16, 17), very little is known about the modulatory characteristics of the various classes of pattern recognition receptors and microbial ligands. The aim of this study was to systematically investigate the potential to either train or induce tolerance of the well-known classes of PRRs expressed on monocytes/macrophages, as depicted in Table 1. We demonstrate that several bacterial, fungal, and viral ligands induce the functional reprogramming of monocytes, leading to either nonspecific enhanced (training) or diminished (tolerance) cytokine production upon secondary stimulation, a process that is often dependent on ligand concentration. Understanding the nature of the signaling pathways in determining the functional fate of innate host responses upon sequential stimulation of pattern recognition receptors may represent an important step toward understanding bacterial and fungal colonization and/or invasion of the mucosa on one hand, and for developing novel immunotherapeutic strategies on the other hand.

**TABLE 1:** Pattern recognition receptors and microbial ligands and their adapter molecules

PRR	Microbial ligands^*a*^	Adapter molecule^*b*^
Membrane receptors		
Dectin-1	β-glucan	CARD9
TLR2	Lipoteichoic acid (Pam3CSK4)	TIRAP
	Lipoproteins	MyD88
	Glycosyl-phosphatidylinositols from Toxoplasma gondii	
TLR4	Lipopolysaccharide	TIRAP
	Envelope proteins of RSV	MyD88
	Glycosyl-phosphatidylinositols from T. gondii	TRAM
		TRIF
TLR5	Flagellin	MyD88
Endosomal receptors		
TLR3	dsRNA	TRIF
	Poly (I·C)	
TLR7/8	ssRNA (R848)	MyD88
TLR9	CpG DNA	MyD88
Cytosolic receptors		
NOD1	Tri-DAP	CARD6
NOD2	Muramyl dipeptide	CARD12

a RSV, respiratory syncytial virus; dsRNA, double-stranded RNA; ssRNA, single-stranded RNA.

b TRAM, Toll-like receptor adaptor molecule.

## MATERIALS AND METHODS

### Blood samples.

Blood samples were collected from healthy volunteers at the Sanquin Blood Supply in Nijmegen, the Netherlands. Informed consent was obtained from the volunteers included in the study. Human peripheral blood mononuclear cells (PBMCs) were isolated from buffy coats.

### Reagents.

The culture medium used was Roswell Park Memorial Institute (RPMI) 1640 Dutch modifications from Sigma-Aldrich, supplemented with 1% gentamicin, 1% l-glutamine, and 1% pyruvate (Life Technologies, Nieuwerkerk, the Netherlands). Candida β-glucan was isolated and purified as previously described (23). Other reagents were obtained as follows: Pam3CSK4 (product code L2000; EMC microcollections), LPS (Escherichia coli serotype 055:B5; Sigma-Aldrich) with an additional purification step (24), flagellins from Salmonella enterica subsp. enterica serovar Typhimurium FLA-ST, CpG type C oligodeoxynucleotide (ODN) M362, l-Ala-γ-d-Glu-mDAP (Tri-DAP), and R848 (InvivoGen), poly(I·C) (Brunswick, USA), Syk inhibitor (catalog no. 574711; EMD Millipore), p38 inhibitor SB-202190 (product no. S7067; Sigma-RBI), extracellular single-regulated kinase (ERK) inhibitor (product no. V1121; Promega), and Jun N-terminal protein kinase (JNK) inhibitor SP600125 (product no. S-2022; AG Scientific, Inc.), phorbol myristate acetate (PMA)(Sigma), CD45-PC7 (Beckman Coulter), CD68-antigen-presenting cell (APC) (BioLegend), CD14-phycoerythrin (PE) (Beckman Coulter), CD11b-fluorescein isothiocyanate (FITC) (Beckman Coulter), and DC-SIGN (BioLegend). Versene solution was purchased from Invitrogen, and the CytoTox nonradioactive assay was purchased from Promega. The Syk inhibitor was used previously, as described (16).

The epigenetic inhibitors, used previously (16, 17), were as follows: histone methyltransferase inhibitor (5′-deoxy-5′-methylthioadenosine [MTA]) (product no. D5011; Sigma-Aldrich), epigallocatechin-3-gallate (EGCG) histone acetyltransferase inhibitor (HAT-i) (product no. E4143; Sigma), histone demethylase inhibitor (pargyline, product no. P8013; Sigma-Aldrich), and histone deacetylase inhibitor ITF2357/9 (Italfarmaco SpA) (25, 26).

### Stimulation experiments.

Mononuclear cells were isolated by density centrifugation of phosphate-buffered saline (PBS)-diluted blood (dilution 1:1) over a Ficoll-Paque Plus gradient (GE Healthcare). PBMCs were washed 3 times with PBS and suspended in RPMI 1640 culture medium supplemented with 1% gentamicin, 1% pyruvate, and 1% l-glutamine. Cells were counted in a Coulter counter (Coulter Electronics), and the concentration was adjusted to 5 × 10^6^ cells/ml. For stimulation experiments, a 100-μl suspension of 5 × 10^5^ PBMCs in RPMI medium was added to flat-bottomed 96-well plates (Greiner Bio-One) and incubated for 1 h at 37°C and 5% CO_2_. The adherent monocytes were selected by washing out nonadherent cells three times with warm PBS. For training, the cells were preincubated for 24 h with either RPMI (negative control), β-glucan (1 μg/ml) (positive control), or serial dilutions of Pam3CSK4 (100 μg/ml to 0.01 pg/ml), LPS (100 ng/ml to 10^−5^ pg/ml), flagellin (10 μg/ml to 0.01 pg/ml), poly(I·C) (100 μg/ml to 0.01 pg/ml), R848 (100 μg/ml to 0.01 pg/ml), CpG (10 μg/ml to 0.001 pg/ml), Tri-DAP (10 μg/ml to 0.001 pg/ml), and muramyl dipeptide (MDP) (10 μg/ml to 0.01 pg/ml) for 24 h. After the first incubation, monocytes were washed with warm PBS and maintained in RPMI supplemented with 10% pooled human blood serum for 5 days (medium was refreshed at day 3). Thereafter, the cells were subjected to a second stimulation of cytokine production with LPS (10 ng/ml), Pam3CSK4 (10 μg/ml), or RPMI (negative control). After 24 h, duplicate supernatants were collected, pooled, and stored at −20°C until assayed.

To investigate whether various inhibitors affect training, the adherent monocytes were preincubated for 1 h prior to the first stimulation with p38 inhibitor (1 μM), Syk inhibitor (140 μM), JNK inhibitor (20 μM), ERK inhibitor (10 μM), MTA (1 mM), EGCG (15 μM), pargyline (3 μM), or ITF2357/9 (100 nM). Subsequently, the microbial stimuli were added to the cells with the inhibitors for an additional 24 h. Thereafter, the cells were washed with PBS and further incubated for 5 days in culture medium supplemented with 10% pooled human serum. On day 6, the trained macrophages were subjected to a second stimulation for 24 h with either LPS or Pam3CSK4. The supernatants were collected and stored at −20°C until assessed.

### Cytokine assay.

The concentrations of tumor necrosis factor alpha (TNF-α) (R&D Systems, Abingdon, United Kingdom) and interleukin 6 (IL-6) (Sanquin, Amsterdam, Netherlands) were measured in cell culture supernatants 24 h after the second stimulation using an enzyme-linked immunosorbent assay (ELISA), according to the manufacturer's instructions.

Lactate hydrogenase (LDH) measurements were performed with the CytoTox 96 nonradioactive cytotoxicity assay (Promega), according to the manufacturer's instructions. Briefly, the adherent monocytes were incubated for 24 h with different microbial ligands, and the secreted LDH was measured in the cell culture supernatants. To check for putative cytotoxic effects of mitogen-activated protein kinase (MAPK), Syk, and epigenetic inhibitors, the adherent monocytes were incubated for 24 h with RPMI, MAPK, Syk, or epigenetic inhibitors. Thereafter, the supernatants and cells were collected and stored until assessed. The values of the inhibitors in RPMI were considered to be background and subsequently subtracted from the final results. RPMI was considered a negative control. The final values are depicted in percentages.

### Extracellular cytokine staining and flow cytometry.

Three milliliters of PBMCs with a concentration of 5 × 10^6^ cells/ml was cultured in a well of a 6-well plate. After 1 h of incubation at 37°C and 5% CO_2_, the nonadherent cells were washed 3 times with warm PBS. The remaining adherent monocytes were prestimulated for 24 h with concentrations of different ligands that would induce either training or tolerance, such as β-glucan (1 μg/ml), MDP (1 μg/ml), flagellin (1 μg/ml), R848 (1 μg/ml), Pam3CSK4 (1 μg/ml), and RPMI (negative control). The stimuli were washed away and the cells were further incubated for 5 days in the presence of RPMI supplemented with 10% pooled human serum. On day 7, the cells were incubated for 1 h with Versene solution, collected, harvested by centrifugation, and suspended in PBS supplemented with 1% protein blocking agent (PBA). The cells were washed two times and stained extracellularly with the following antibodies: anti-CD45-PeCy7 (Beckman Coulter), anti-CD68-APC (BioLegend), anti-CD14-PE (Beckman Coulter), anti-CD11b-FITC (Beckman Coulter), and anti-DC-SIGN-APC (BioLegend). The samples were measured on a fluorescence-activated cell sorter (FACS) FC500, and the data were analyzed using the CXP software (Beckman Coulter).

### Statistical analysis.

IBM SPSS Statistics software, version 20, was used to perform statistical analysis. All experiments were performed at least three times, with a minimum of six volunteers. In order to assess the training effect of a specific ligand upon a secondary stimulation, nontrained cells stimulated with LPS (or Pam3CSK4) were compared with trained cells restimulated with LPS (or Pam3CSK4). Differences between the groups were analyzed using the Wilcoxon signed-rank test and were considered statistically significant at a *P* value of <0.05.

## RESULTS

### Type of ligand-PRR interactions and pathogen-associated molecular pattern concentration decides the inflammatory state of monocytes.

**FIG 1:**
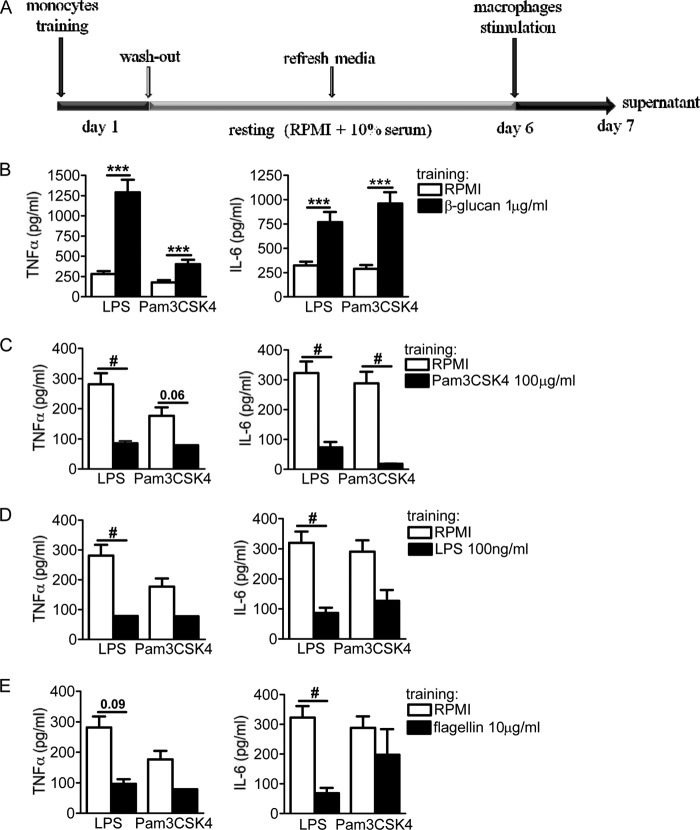
(A) *In vitro* training scheme. Adherent monocytes were incubated with pure ligands (training stimuli) for 24 h at 37°C. Following the first incubation, the cells were washed with warm PBS and further incubated with RPMI and pooled human serum for 5 days. In the absence of any training stimulus during the first 24 h, primary monocytes maintained in culture for several days eventually differentiated into macrophages. After this resting period, different pattern recognition receptor ligands were added (second stimulus) for an additional 24 h. (B to E) Tolerance induced by the membrane receptors. The cells were preexposed for 24 h to culture medium, β-glucan (1 μg/ml) (B), Pam3CSK4 (100 μg/ml) (C), LPS (100 ng/ml) (D), or flagellin (10 μg/ml) (E). Primary stimuli were washed out, and after 6 days, the macrophages were restimulated with RPMI (negative control), LPS (10 ng/ml), or Pam3CSK4 (10 μg/ml). While β-glucan induced training, Pam3CSK4, LPS, and flagellin induced tolerance. The data are presented as means ± standard error of the mean (SEM) (*n* > 8, from 5 independent experiments). *, *P* < 0.05; **, *P* < 0.01; ***, *P* < 0.001; #, *P* < 0.05 (tolerance). The Wilcoxon signed-rank test was used to detect significant differences.

Some microbial ligands, such as β-1,3-glucan (β-glucan) and the muramyl dipeptide (MDP) component of peptidoglycans, induce trained immunity through the engagement of dectin-1 and nucleotide-binding oligomerization domain 2 (NOD2), respectively (16, 17), while the TLR4 agonist, LPS, induces tolerance (19, 20). We performed a systematic assessment to determine whether exposure of adherent monocytes to the microbial ligands of the TLR and NOD-like receptor (NLR) classes of pattern recognition receptors, such as Pam3CSK4, LPS, flagellin, poly(I·C), R848, CpG, and Tri-DAP, also influences the cell response to a secondary stimulation. The adherent monocytes were therefore exposed to each different PRR ligand for 24 h (first stimulus) at a concentration that would induce an inflammatory response, followed by a washout step. Following a resting time of 5 days, a period during which monocytes in the absence of any primary event differentiate into macrophages, the cells were exposed to a secondary stimulation with either LPS or Pam3CSK4 (second stimulus) (Fig. 1A).

**FIG 2:**
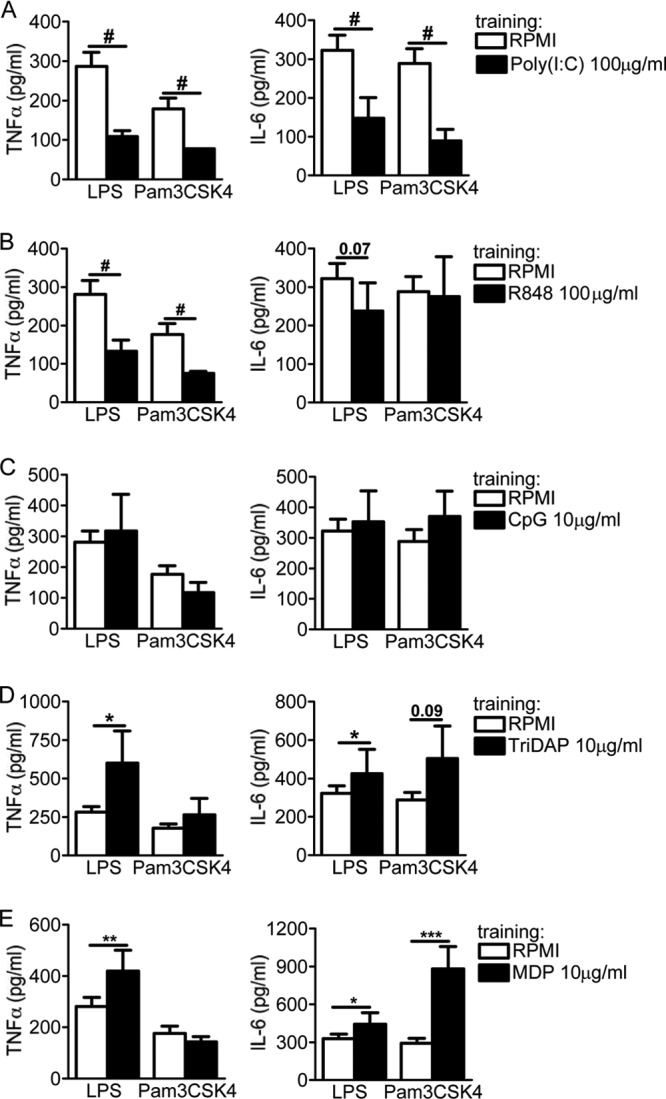
Differential effects of endosomal and cytosolic receptors on adherent monocytes. The cells were preexposed for 24 h to culture medium, poly(I·C) (100 μg/ml) (A), R848 (100 μg/ml) (B), CpG (10 μg/ml) (C), Tri-DAP (10 μg/ml) (D), and MDP (10 μg/ml) (E). Primary stimuli were washed out, and after 6 days, the macrophages were restimulated with RPMI (negative control), LPS (10 ng/ml), or Pam3CSK4 (10 μg/ml). While poly(I·C) induced tolerance for TNF-α and IL-6, R848 was able to induce tolerance for TNF-α only, and CpG did not exert any significant effect on the inflammatory status of monocytes. Both types of NLRs were able to enhance the status of monocytes upon a second exposure to either LPS or Pam3CSK4. The data are presented as the means ± SEM (*n* > 8, from 5 independent experiments). *, *P* < 0.05; **, *P* < 0.01; ***, *P* < 0.001; #, *P* < 0.05 (tolerance). The Wilcoxon signed-rank test was used to detect significant differences.

The Candida albicans cell wall component β-glucan strongly trains monocytes, leading to an enhanced production of TNF-α and IL-6 upon nonspecific secondary stimulation with LPS or Pam3CSK4 (16); therefore, β-glucan preincubation of monocytes was used as an inherent control of effective training throughout this study (Fig. 1B). In contrast to the training obtained upon engagement of dectin-1, the stimulation of the membrane receptors TLR2, TLR4, and TLR5 by inflammatory doses of Pam3CSK4 (100 μg/ml), LPS (100 ng/ml), and flagellin (10 μg/ml) induced a long-term tolerant state in which monocytes produced fewer proinflammatory cytokines (TNF-α and IL-6) upon restimulation than did the RPMI-treated control cells (Fig. 1C to E). Although LPS tolerance has been described *in vitro* (21) and *in vivo* (27) in short-term experiments, no studies have assessed the long-term effects of LPS. Using our training model of human adherent monocytes, we described a long-term tolerance effect of LPS (100 ng/ml) on the cytokine production observed upon secondary stimulation. In contrast to the membrane-associated TLRs, engaging cytosolic PRRs during the primary exposure to pure ligands revealed more diverse outcomes in terms of innate immune memory features. Preactivation of the endosomal TLR3 with a strong inflammatory dose of poly(I·C) (100 μg/ml) maintained the cells in a tolerant status, being refractory to the second stimulation (Fig. 2A). Similarly, engagement of the endosomal TLR7/8 with R848 (100 μg/ml) also induced a refractory state of monocytes for TNF-α but not for IL-6 (Fig. 2B). However, the use of CpG (10 μg/ml) for preactivating TLR9 failed to induce any trained or tolerant effect (Fig. 2C). In contrast, the engagement of NOD1 by Tri-DAP (10 μg/ml) and NOD2 by MDP (10 μg/ml) significantly trained the cells toward an increased production of TNF-α and IL-6 (Fig. 2D and E). Of note, while no correlation was observed between the trained or refractory status obtained and the intracellular adapter molecules of TLRs (i.e., MyD88, Toll-interleukin 1 receptor [TIR] domain-containing adaptor protein [TIRAP], and TIR domain-containing adapter-inducing beta interferon [TRIF]) (Table 1), it seemed that engaging receptors that signal through caspase recruitment domain (CARD) molecules favored an enhanced trained immunity status. Altogether, the activation of TLRs present at the cell surface seemed to maintain the cells in a refractory status. However, engaging endosomal TLRs had a striking and different effect on the functional fate of monocytes, regardless of the adaptor molecules that were used to signal. Finally, engaging cytosolic NLRs seemed to result in an enhanced proinflammatory immune status.

**FIG 3:**
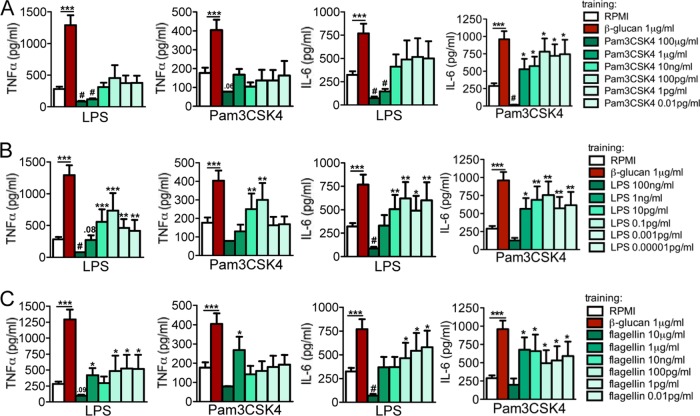
Dose responses of membrane-bound receptors. The cells were preexposed for 24 h to culture medium or to different concentrations of Pam3CSK4 (100 μg/ml to 0.01 pg/ml) (A), LPS (100 ng/ml to 10^−5^ pg/ml) (B), and flagellin (10 μg/ml to 0.01 pg/ml) (C). The first stimuli were washed away, and adherent monocytes were further incubated for 5 days in culture medium supplemented with 10% pooled human pool serum. At day 6, the cells were restimulated with RPMI (negative control), LPS (10 ng/ml), or Pam3CKS4 (10 μg/ml). Depending on the concentration used, after several days, cells entered either a trained or tolerized status. The data are presented as the means ± SEM (*n* > 8, from 5 independent experiments), *, *P* < 0.05; **, *P* < 0.01; ***, *P* < 0.001; #, *P* < 0.05 (tolerance). The Wilcoxon signed-rank test was used to detect significant differences. TNF-α and IL-6 from cells stimulated with RPMI were below the detection limit.

In order to assess whether the ligand concentration influences the training of monocytes, titrations of all the ligands assessed in this study were performed. None of the first incubations with low doses of ligands induced measurable amounts of proinflammatory cytokines in the supernatants collected after the first 24 h (data not shown). We first deciphered the titration response of the cell membrane-associated TLRs. Systematically lowering the amount of Pam3CSK4, which is used to activate the cell membrane-associated TLR2, strongly modified the monocyte reprogramming characteristics. Pam3CSK4 in a low concentration (1 μg/ml) resulted in a tolerance status for TNF-α and IL-6 upon LPS secondary stimulation. Interestingly, upon restimulation with Pam3CSK4, Pam3CSK4-preactivated monocytes were programmed to produce an increased amount of IL-6 compared to that produced by nonactivated (RPMI) control cells (Fig. 3A). The anti-inflammatory-trained pattern was generally observable for TNF-α upon LPS secondary stimulation (Fig. 3A). Similarly, decreasing the amount of LPS and flagellin during the preactivation period of monocytes led to the opposite effect of tolerance, which is a proinflammatory status represented by significantly elevated levels of TNF-α and IL-6 upon restimulation (Fig. 3B and C).

**FIG 4:**
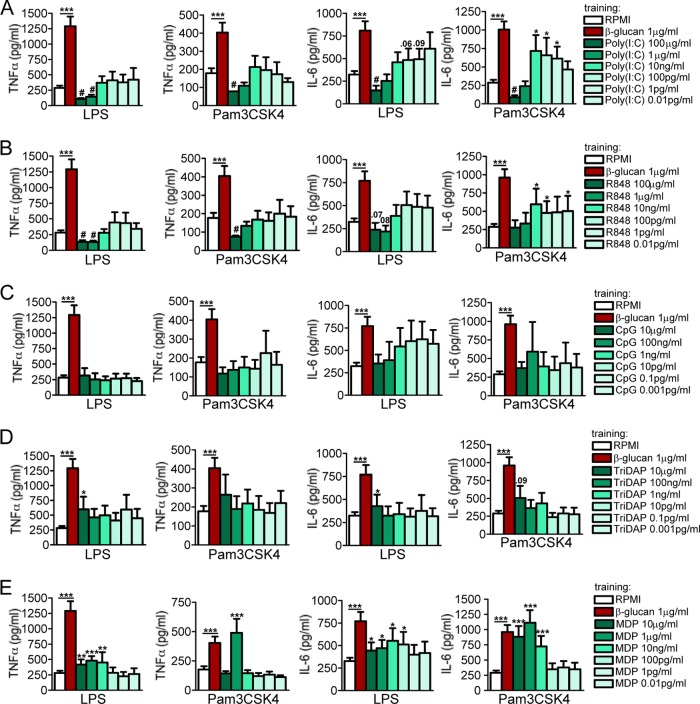
Dose responses of endosomal and cytosolic receptors. The cells were preexposed for 24 h to culture medium or to different concentrations of poly(I·C) (100 μg/ml to 0.01 pg/ml) (A), R848 (100 μg/ml to 0.01 pg/ml) (B), CpG (10 μg/ml to 0.001 pg/ml) (C), Tri-DAP (10 μg/ml to 0.001 pg/ml) (D), and MDP (10 μg/ml to 0.01 pg/ml) (E). The first stimuli were washed away, and adherent monocytes were further incubated for 5 days in culture medium supplemented with 10% pooled human pool serum. At day 6, the cells were restimulated with RPMI (negative control), LPS (10 ng/ml), or Pam3CKS4 (10 μg/ml). Depending on concentration used, after several days, cells entered either a trained or tolerized status. The data are presented as the means ± SEM (*n* > 8, from 5 independent experiments), *, *P* < 0.05; **, *P* < 0.01; ***, *P* < 0.001; #, *P* < 0.05 (tolerance). Wilcoxon signed-rank test was used to detect significant differences. TNF-α and IL-6 from cells stimulated with RPMI were below the detection limit.

We next deciphered the effect of differential engagement of cytosolic receptors. Preincubation of cells with low doses of poly(I·C) or R848 gradually reversed the long-term tolerance effect (Fig. 4A and B), leading to a training effect upon restimulation with LPS or Pam3CSK4. Interestingly, lowering the concentrations of CpG (Fig. 4C), Tri-DAP (Fig. 4D), or MDP (Fig. 4E) did not reverse the long-term effect obtained with high inflammatory doses. Particularly, lowering the doses of CpG did not alter the “inert” status of priming, and none of the doses used triggered an enhanced or refractory immune status upon restimulation of the monocytes (Fig. 4C). However, although the enhanced proinflammatory status of the monocytes was not reversed to tolerance, the training effect obtained with the preactivation of NLRs eventually vanished by lowering the amount of Tri-DAP and MDP, resulting in normal nontolerant and nontrained cells (Fig. 4D and E). Cell viability and ligand toxicity were assessed at 24 h and at 7 days after the first stimulation for all the ligands tested, and none of the ligands used during the 24 h of primary cell stimulation enhanced LDH release compared to RPMI-treated cells (see Fig. S1 in the supplemental material), demonstrating that the decreased cytokine production was not due to the toxicity of the ligands used in the experiments.

### Trained or tolerized cells exhibit a broad range of cell size, granulocity, and different macrophage markers at the surface.

**FIG 5:**
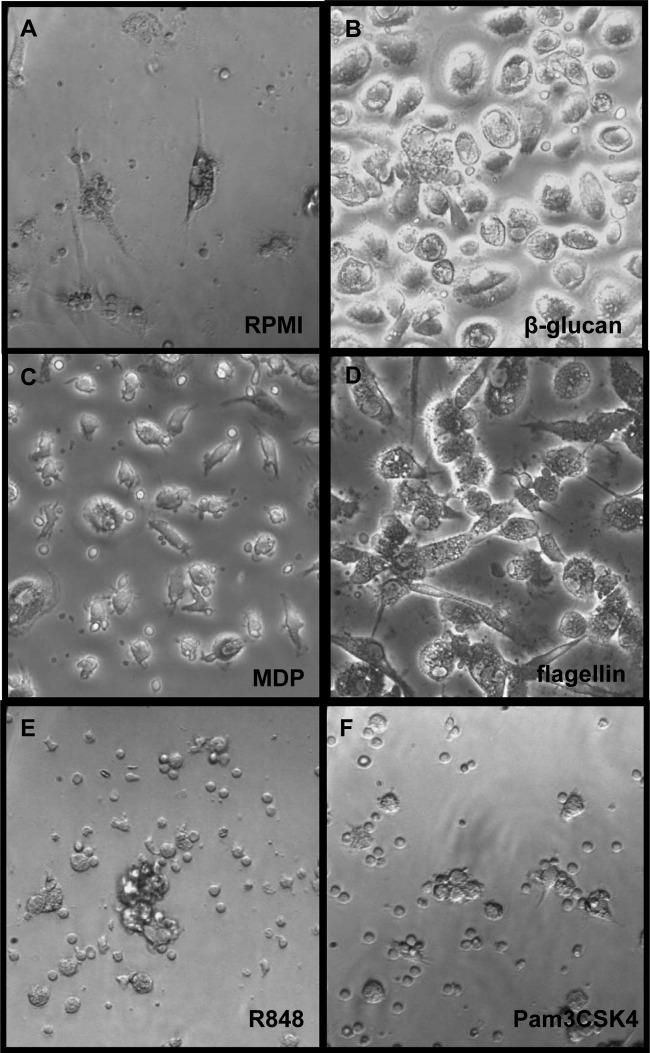
Morphology of trained versus tolerized cells. The cells were preexposed for 24 h to culture medium (A) or to several pure ligands, such as β-glucan (1 μg/ml) (B), MDP (1 μg/ml) (C), flagellin (1 μg/ml) (D), R848 (1 μg/ml) (E), and Pam3CSK4 (1 μg/ml) (F). The first stimuli were washed away, and adherent monocytes were further incubated for 5 days in culture medium supplemented with 10% pooled human pool serum. At day 6, the cells were microscopically examined and representative pictures were chosen (×200). After several days in culture, primary monocytes differentiated into the macrophages. While trained cells appeared to be bigger and more activated (B, to D), cells that entered a tolerizing status seemed to be considerably smaller (E and F).

Macroscopically, cells trained with β-glucan (1 μg/ml), MDP (1 μg/ml), and flagellin (1 μg/ml) are considerably larger than nontreated cells (Fig. 5A to D), while macrophages that entered a tolerized status were comparable in size to RPMI-treated cells (Fig. 5E and F). The phenotype of considerably larger trained cells is reminiscent of a previous study by Daigneault et al. (28) in which human monocyte-derived macrophages with serum and PMA-activated-THP1 cells via activation showed increased light scattering due to the accumulation of organelles (28). To establish the similarities and differences between long-term innate immune reprogramming by PRR ligands and the process of PMA-induced cell activation, we assessed the effect of two different concentrations of PMA (10 μg/ml and 10 ng/ml) in our *in vitro* experimental model. Preincubating monocytes with a low dose of PMA (10 ng/ml) during the first 24 h resulted in an enhanced proinflammatory status, while the higher concentration (10 μg/ml) induced a refractory immune status compared to the nonactivated control macrophages (see Fig. S2 in the supplemental material). Hence, these results suggest the ability of PMA to induce differential activation of monocytes at the level of proinflammatory cytokine production and in a dose-dependent manner, similarly to TLR ligands.

**FIG 6:**
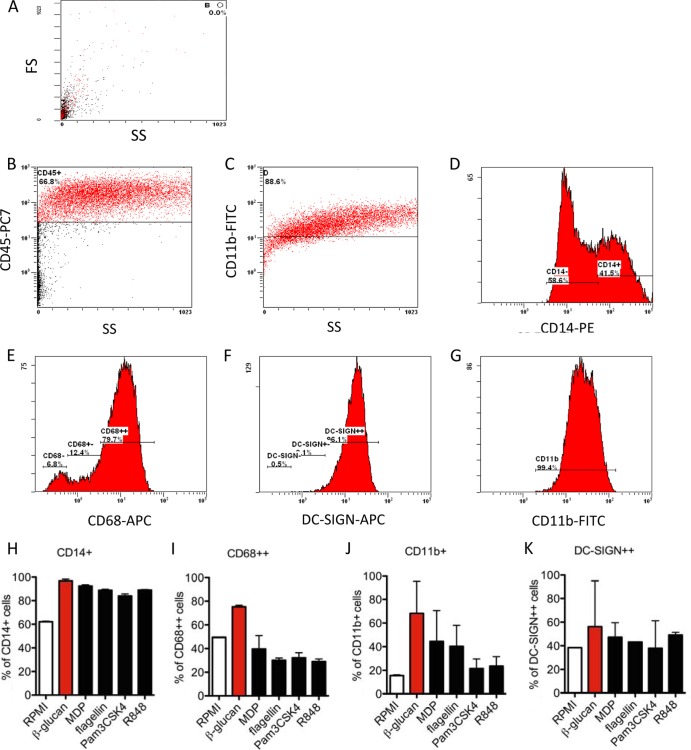
Flow cytometric analysis of CD14-, CD68-, DC-SIGN-, and CD11b-positive cells gated on CD45^+^ populations after prestimulation with β-glucan, MDP, flagellin, R848, and Pam3CSK4. A total of 3 ml at 5 × 10^6^ PBMCs was incubated for 1 h, washed 3 times with PBS, and the remaining adherent monocytes were coincubated with β-glucan (1 μg/ml), MDP (1 μg/ml), flagellin (1 μg/ml), R848 (1 μg/ml), and Pam3CSK4 (1 μg/ml), or with RPMI (untrained control) for 24 h. Thereafter, the cells were washed and further incubated with RPMI supplemented with 10% pooled human serum for 5 days. At day 6, the macrophages were incubated with Versene solution for 1 h at 37°C, detached from the plate, and stained extracellularly using anti-CD45 PECy7, anti-CD14 PE, anti-CD68 APC, anti-DC-SIGN APC, and anti-CD11b FITC antibody. (A) The side scatter (SS) and forward scatter (FS) of the RPMI-treated cells are shown. (B and C) One representative picture of the staining and gating on CD45^+^ high β-glucan-trained cells is shown. Depending on the fluorescence intensity as well as cell size, we identified several cell populations, depicted as negative (−), positive (+), and high positive (++). Gating on different populations of CD14^+^ β-glucan-trained cells (D), CD68^+^ β-glucan-trained cells (E), DC-SIGN^+^ β-glucan-trained cells (F), and CD11b^+^ β-glucan-trained cells (G) are shown. (H to K) % of positive cells gated on CD45^+^ high. The data are presented as the means ± SEM (*n* > 2, from 4 independent experiments).

To further investigate the phenotype of trained versus tolerant cells, we assessed several surface markers on the cells treated with different pathogen-associated molecular patterns (PAMPs) by means of flow cytometry (Fig. 6). Adherent monocytes were trained as previously described (Fig. 1A), and after 6 days, cells were restimulated with medium alone. All the counts were gated on CD45^+^ high cells (Fig. 6B and C). β-glucan was used as an inherent control; thus, based on β-glucan-trained macrophages, several cell populations were identified for CD14^+^, CD68^+^, and DC-SIGN^+^ cells (Fig. 6D to F), while for CD11b^+^ cells, only one cell population was identified (Fig. 6G). The levels of CD14^+^, CD68^+^, DC-SIGN^+^, and CD11b^+^ cells at the cell surface were assessed. Interestingly, based on the side and forward scatter analyses, all β-glucan-trained cells proved to be of a broad granulocity and size range, and most of the cells were positive for CD14, CD68, and CD11b (Fig. 6H to J). Similar observations were obtained with MDP- and flagellin-treated cells at a concentration that induces training (1 μg/ml) (Fig. 6H and J), while CD68^+^ and DC-SIGN^+^ cells were less present among the MDP- and flagellin-trained cells. Tolerant macrophages treated with Pam3CSK4 (1 μg/ml) or R848 (1 μg/ml) were significantly smaller than β-glucan-trained cells, with a broad granulocity, and fewer were positive for CD68, CD11b, or DC-SIGN, while CD14 was still present to a similar intensity as β-glucan-trained cells (Fig. 6H to K).

### Trained immunity is dependent on MAP kinase-dependent pathways and histone methylation and acetylation.

**FIG 7:**
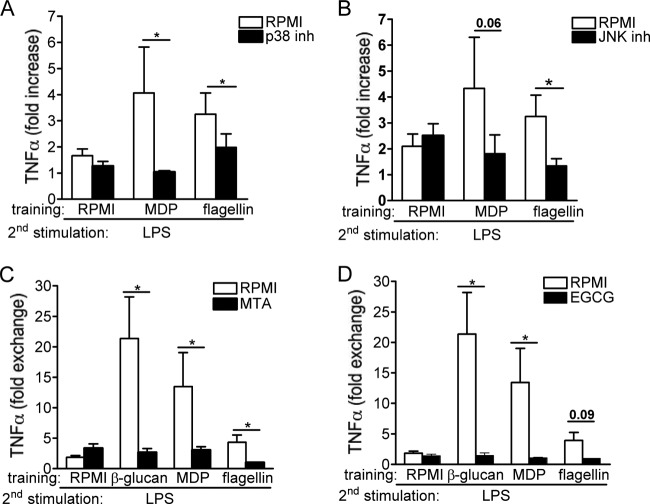
(A and B) MAP kinase-dependent pathways mediate trained immunity. Adherent monocytes were preincubated for 1 h with culture medium, p38, ERK, JNK, or Syk inhibitor (inh). After this inhibition period, 100 μl per well of either culture medium, flagellin (1 μg/ml), or MPD (1 μg/ml) was added to the cell suspension, without removing the inhibitors, for 24 h. Thereafter, the cells were washed and incubated with RPMI and 10% pooled human serum. At day 6, the cells were subjected to a second stimulation with LPS (10 ng/ml) for an additional 24 h. The data are presented as the means ± SEM (*n* ≥ 7, from four independent experiments). *, *P* <0.05. The Wilcoxon signed-rank test was used to detect significant differences. (C and D) Blocking histone methylation and acetylation inhibits trained immunity induced by MDP and flagellin. Before training, adherent monocytes were preincubated for 1 h with culture medium or with different epigenetic inhibitors, such as histone methyltransferase inhibitor (MTA), histone acetyltransferase inhibitor (EGCG), histone demethylase inhibitor (pargyline), and the histone deacetylase inhibitor ITF2357/9. Thereafter, 100 μl of culture medium, β-glucan (10 μg/ml), flagellin (1 μg/ml), or MPD (1 μg/ml) was added to the cell suspension, without removing the inhibitors, and further incubated for an additional 24 h. At day 6, the macrophages were subjected to a second stimulation with LPS (10 ng/ml) for another 24 h. The data are presented as the means ± SEM (*n* = 6, from three independent experiments), *, *P* < 0.05. The Wilcoxon signed-rank test was used to detect significant differences.

MAP kinases (MAPK) play a key role in the induction of the initial inflammatory cytokine response of the activated monocytes (29). This prompted us to investigate whether MAPK (ERK, JNK, and p38) and Syk pathways are involved in the training effects of monocytes. While β-glucan signaling induces trained immunity via the noncanonical Raf1 pathway (16), we show here that p38 and JNK are involved in the training of primary monocytes by MDP and flagellin (Fig. 7A and B). In contrast, ERK and Syk inhibitors did not affect training by these ligands (data not shown). β-glucan- and Mycobacterium bovis BCG-induced trained immunity is mediated by epigenetic mechanisms (16, 17). We assessed whether histone modifications by methylation or acetylation influence the observed training effects of MDP and flagellin. The inhibition of histone methyltransferases with 5′-deoxy-5′-methylthioadenosine (MTA) or inhibition of histone acetyltransferases with epigallocatechin-3-gallate (EGCG) drastically inhibited the training of monocytes (Fig. 7C and D). In contrast, the inhibitors of histone demethylase or deacetylase enzymes did not significantly influence the training effects of MDP or flagellin. Notably, the putative cytotoxic effects of MAPK, Syk, and epigenetic inhibitors used in the study were assessed by LDH measurements. None of the inhibitors used during the 24 h of primary cell stimulation enhanced LDH release compared with RPMI-treated cells (see Fig. S3 in the supplemental material), demonstrating that the molecules were not toxic to the cells.

## DISCUSSION

In the present study, we show that primary exposure to microbial ligands alters the functional fate of monocytes, which is determined by the nature and concentrations of the PRRs engaged. Engagement of NLRs (NOD2 or NOD1 receptors) induces trained immunity, a long-term enhanced immune status upon priming with high doses of MDP or Tri-DAP, which can vanish with smaller amounts of ligands. The engagement of TLRs with high inflammatory doses of PRR ligands predominantly induces tolerance, with the exception of CpG, for which tolerance seems to be inherent at any dose assessed. Interestingly, low concentrations of TLR ligands not only abolished the tolerance-induced effect but reversed it, and in fact, trained the monocytes to be maintained in a heightened proinflammatory state. Furthermore, functional reprogramming of monocytes was also associated with cell shape and cell surface marker modifications. Finally, training of monocytes by MDP and flagellin is mediated by p38 and JNK MAPK-dependent pathways and depends on epigenetic histone modifications.

Earlier studies have shown that β-glucan recognition by monocytes through dectin-1 and CR3 induces trained immunity (30), which mediates protection to reinfection in a T-/B-cell-independent fashion (16). Similarly, BCG vaccination in human volunteers induces epigenetic reprogramming of monocytes and enhances secondary responses to microbial stimulation (17). In this study, we report that differential engagement of PRRs and that PMA activation of monocytes might result in either an enhanced or refractory innate immune status. These findings raise an important conceptual question: what is the difference between a stimulated cell, a differentiated macrophage, and a trained immune cell? The difference can be defined at a molecular epigenetic level: the unstimulated monocyte or macrophage is characterized by the absence of cytokine production, with silent markers for both histone acetylation and methylation. During cell activation and active transcription, both histone methylation and histone acetylation are present at the promoter and enhancer levels, potentiating gene transcription. The trained cell has lost its acetylation markers and thus has lost active transcription (after an initial stimulation). However, in a trained cell (in contrast to a naive cell), the promoters of inflammatory genes are tagged by histone methylation, which allows for an accelerated and stronger response upon restimulation. We recently described the H3K4 methylation as a property of trained monocytes (16), and this characteristic was confirmed in a landmark study by Ostuni et al. (31) showing the kinetic events during macrophage training. The authors proposed the name “latent enhancers” for the epigenetic units determining cell training (31). The opposing process of trained immunity is the innate immune tolerance, which has been known for more than half a century and which was recently demonstrated to be regulated by epigenetic events (21, 32, 33).

Despite the importance of innate immune training and tolerance, no systematic studies have attempted to define which microbial ligands and pattern recognition receptors induce trained immunity or tolerance. In the present study, we screened the major TLR and NLR microbial ligands for their capacity to induce either training or tolerance. Although dose dependent, we identified important bacterial ligands, such as flagellin and MDP, which can induce trained immunity. In flagellated bacteria, flagellin is one of the major proteins in the cell, and mammals tend to have a strong immune response to flagellin. This molecule is recognized in mammals by Toll-like receptor 5 (34, 35). Immune responses to flagellin have been proposed to play an important role in the pathogenesis of inflammatory bowel diseases (36, 37). From this perspective, the capacity of flagellin to induce trained immunity and a more potent inflammatory response may have important consequences for the pathogenesis of this disease. On the other hand, our data may lead to the conclusion that flagellin represents an important new type of adjuvant for improving vaccination, a hypothesis that is supported by earlier studies showing that flagellin is an effective adjuvant for immunization against lethal respiratory challenge with Yersinia pestis (38). Muramyl dipeptide is a component of bacterial cell wall peptidoglycan that is recognized by NOD2 (39), which is present in most bacterial species. The importance of NOD2 in protection against inflammatory bowel disease has been highlighted by the fact that particular mutations in NOD2 are associated with susceptibility to Crohn's disease (40, 41). Recently, it has been proposed that MDP functions as a mucosal adjuvant that enhances the immunogenicity of virus-like particles (42). Consequently, its capacity to induce trained immunity may represent an important advantage in these settings.

In contrast, other microbial ligands, such as the TLR9 agonist CpG, had little, if any, long-term effects on the function of monocytes. This argues that only certain ligands at certain concentrations are capable of inducing trained immunity. A particular interesting finding is that TLRs in high doses, with the exception of TLR9, consistently induced long-term tolerance to secondary stimulation with bacterial ligands. These findings are supported by earlier studies showing that the *in vitro* tolerance and cross-tolerance phenomena might be induced via TLR2, TLR4, and TLR9 (43). Additionally, it has been reported that TLR7/8 engagement mediates short-term tolerance in mice both *in vitro* and *in vivo* (44, 45). Further investigation of the mechanisms mediating this effect is warranted, especially in light of the known immunosuppressive effects of viral infections (46, 47). The levels of several activation markers at the cell surface were assessed. Based on the side and forward scatter analyses, β-glucan-, MDP-, and flagellin-trained cells are of a broad granulocity and cell size and mainly positive for CD14 and CD11b, while cells positive for CD68 and DC-SIGN were less present. Tolerant macrophages, treated with Pam3CSK4 or R848, were significantly smaller than β-glucan-trained cells, and fewer were positive for CD68, CD11b, or DC-SIGN, while the activation marker CD14 was still present on these cells. No clear effect was seen on the expression of DC-SIGN on the macrophages. The increase or decrease in these activation markers might thus contribute to an increase or decrease, respectively, in the innate immune responses during the induction of trained immunity; nonetheless, future studies are warranted in order to prove this. In addition, one may also speculate that trained cells present different classes of activation markers than tolerized cells, depending on the subclasses of macrophages induced upon prestimulation.

An important question regarding trained immunity refers to the signaling and molecular mechanisms responsible for its induction. MAP kinases, such as p38, JNK, or ERK, are known to be crucial signaling pathways mediating the stimulation of inflammatory mediators (48, 49), with the p38MAPKα pathway being critical for normal inflammatory responses *in vivo* (50). Notably, the TRIF-dependent late-phase activation of the p38MAPK/MK2 pathway has been demonstrated to be essential for the translational control of TNF-α production (51). LPS regulates the transcript stability of TNF-α (52, 53) via activation of the p38MAPK/MK2 pathway. Considering these crucial biological functions of MAPKs, we assessed their role in the induction of trained immunity. Our data demonstrate that JNK and p38 MAPK are central for the induction of trained immunity by flagellin and MDP, and they might represent potential therapeutic targets for clinical situations in which trained immunity mediates a long-term inflammatory response. In contrast, ERK and Syk do not seem to be needed to elicit trained immunity.

Dynamic chromatin changes across immunological pathways participate in the training mechanism induced by the preincubation of monocytes with β-glucan (16). Exposure of monocytes to β-glucans induces high levels of H3K4 trimethylation at the promoter level of inflammatory genes, which correlates with long-term increased production of proinflammatory cytokines in β-glucan-trained monocytes, a hallmark of trained immunity (16). The blockade of histone methylation or histone acetylation inhibits trained immunity; the first of these modifications has been associated with a state of transcriptional priming, while the latter is associated with actual mRNA transcription. Similar mechanisms of epigenetic memory have been demonstrated in plants during the phenomenon of systemic acquired resistance (54) and may be involved in the resistance to reinfection in invertebrates (55); both classes of organisms are devoid of adaptive immune responses. Thus, the present study demonstrates that even in the presence of adaptive immunity, the training of innate immunity is operational and serves to enhance resistance to certain types of infection.

A key aspect for consideration is that of the duration of trained immunity effects *in vivo* and their potential impact on hematopoietic stem cells. We recently demonstrated trained immunity effects of BCG on monocytes for up to 3 months after vaccination (17). Considering the fact that circulating monocytes have a short half-life of only days, this implies training effects of vaccination on monocyte progenitor cells, and future studies are warranted to assess this important aspect. Moreover, future studies should also assess whether the direct contact of monocytes with microbial ligands is absolutely necessary for the induction of trained immunity, or whether soluble factors, such as cytokines, may also induce similar effects in certain circumstances. In addition, one should also keep in mind that classical monocyte differentiation into macrophages also implies epigenetic changes. However, there are fundamental differences between the epigenetic elements in differentiation versus training of monocytes: in the classical differentiation, both histone methylation and acetylation epigenetic markers lead to the expression of proteins that are characteristic of a macrophage; in the training state, histone acetylation markers are lost, and the promoters of certain genes retain only the histone methylation markers characteristic of latent enhancers (31). These latent enhancers will determine the increased transcription of those genes only after cell stimulation. It can be also envisaged that in certain situations, both training and differentiation markers can be induced, with their respective functional consequences.

Our data have a number of broad implications. By inducing long-term changes in cell capacity to respond to different pathogens, the processes of trained immunity and tolerance might have important effects on the susceptibility of a host to infections. Subsequently, a logical possibility is that the microorganisms encountered by the host on a regular basis may serve to differentiate and continually renew a pool of memory-like macrophages that have enhanced responses to an infectious challenge. By identifying the receptors and signaling pathways that determine the functional fates of monocytes and macrophages, our findings have the potential to lead to the development of new therapeutics that can harness both the potential of trained immunity to induce robust responses to enhance pathogen defenses and of immune tolerance to inhibit autoimmune phenomena.

## Supplementary Material

Supplemental material
