# Supplementary material for: Trained Immunity or Tolerance: Opposing Functional Programs Induced in Human Monocytes after Engagement of Various Pattern Recognition Receptors
Source: Clin Vaccine Immunol. 2014 Apr;21(4):534–45. doi: 10.1128/CVI.00688-13 (PMC3993125; doi:10.1128/CVI.00688-13)
Supplement: Supplemental material [file supp_21_4_534__index.html]

Trained Immunity or Tolerance: Opposing Functional Programs Induced in Human Monocytes after Engagement of Various Pattern Recognition Receptors — Supplemental material 

# Trained Immunity or Tolerance: Opposing Functional Programs Induced in Human Monocytes after Engagement of Various Pattern Recognition Receptors

## Supplemental material

**Files in this Data Supplement:**

- Supplemental file 1 -

  Fig. S1. LDH cytotoxicity assay for all the ligands. Fig. S2. Influence of PMA on prestimulation of primary monocytes. Fig. S3. LDH cytotoxicity assay for the inhibitors.

  PDF, 2.0M
