# Supplementary material for: Trained Immunity or Tolerance: Opposing Functional Programs Induced in Human Monocytes after Engagement of Various Pattern Recognition Receptors
Source: Clin Vaccine Immunol. 2014 Apr;21(4):534–45. doi: 10.1128/CVI.00688-13 (PMC3993125; doi:10.1128/CVI.00688-13)
Supplement: Supplemental material [file CVI.00688-13_zcd999094955so1.pdf]

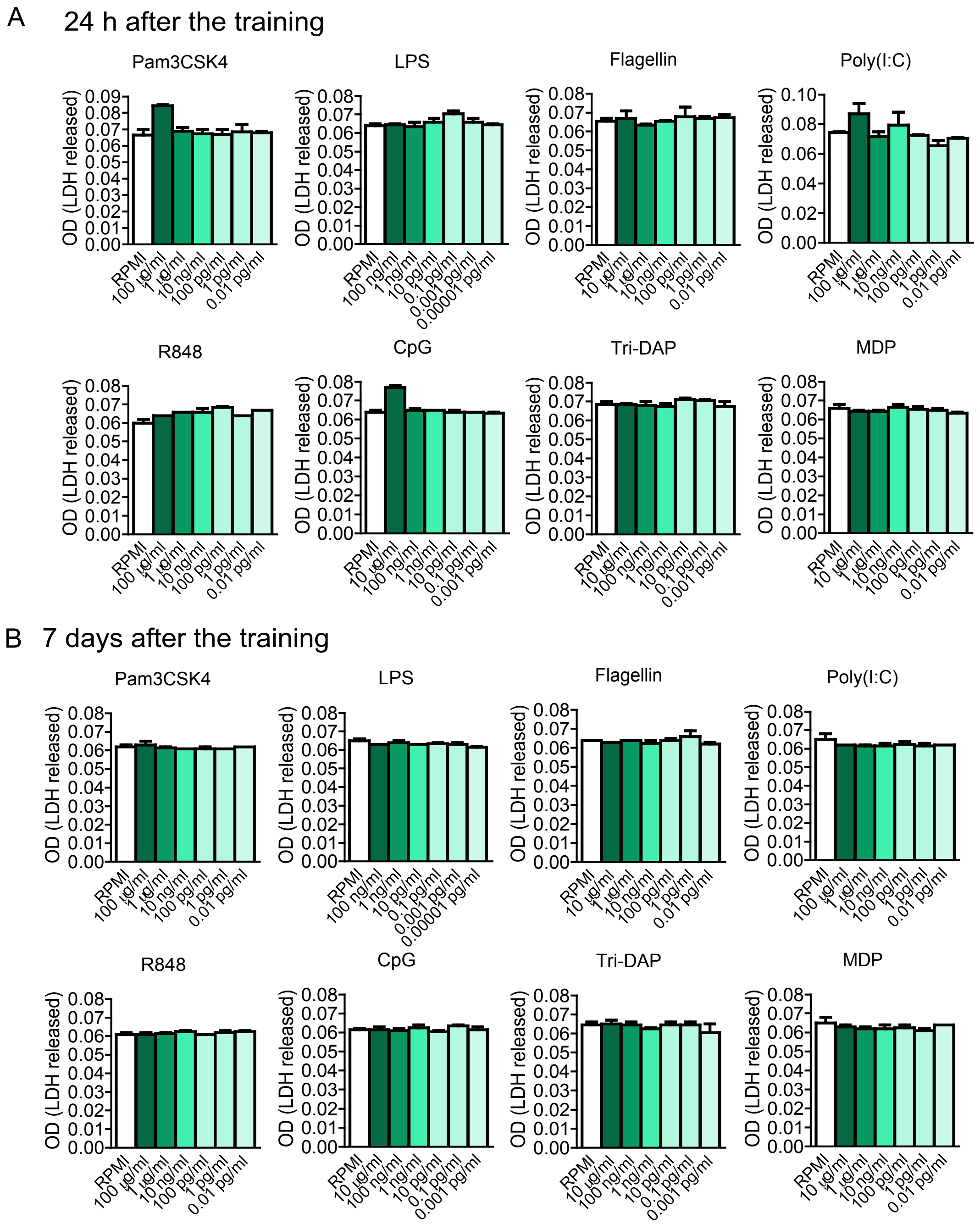

LDH cytotoxicity assay. Adherent monocytes were cocultured for 24 h with either culture medium or with different microbial ligands. Thereafter, supernatants were collected (A) and cells were further cultured in RPMI supplemented with serum for another 6 days. At day 7 supernatants were once again collected (B) and subjected to a cytotoxicity assay, conform to manufacturer’s instructions. All the ligands, independently on the concentration, were proven to be non-toxic to the cells. Data are presented as mean ± SEM (n = 2).

Supplementary Fig. 1

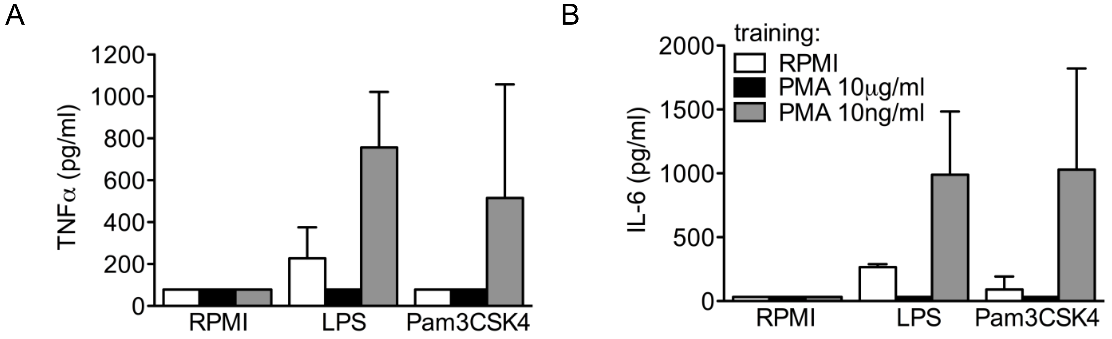

The influence of PMA on prestimulation of primary monocytes. Cells were pre-exposed for 24 h to culture medium or to PMA at two different concentrations: 10 µg/ml and 10 ng/ml. The first stimuli were washed away and adherent monocytes were further incubated for 5 days in culture medium supplemented with 10% pooled human serum. During the 6 days of incubation period, monocytes differentiate into macrophages. Cells were further subjected to a second stimulation with LPS (10 ng/ml) or Pam3CSK4 (10 µg/ml) for an additional 24 h. TNFα (A) and IL-6 (B) were assessed in the cells culture supernatants. Data are presented as mean ± SEM (n = 3).

Supplementary Fig. 2

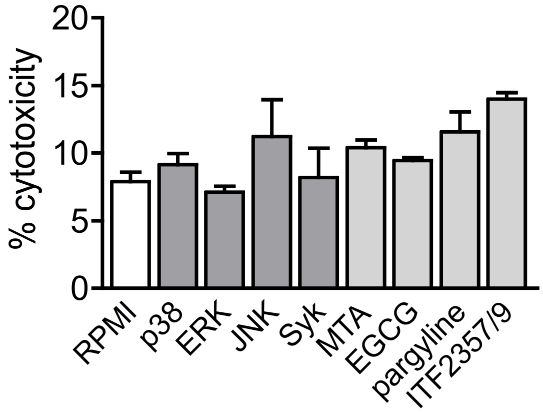

LDH cytotoxicity assay. Adherent monocytes were cocultured for 24 h with either culture medium or with p38-, ERK-, JNK-, Syk- and epigenetic inhibitors (MTA, EGCG, pargyline and ITF2357/9). Thereafter supernatants, as well as cells lysates, were collected and subjected to a cytotoxicity assay, conform to manufacturer's instructions. MAPK-, Syk- as well as epigenetic inhibitors were proven to be non-toxic to the cells. Data are presented as mean ± SEM (n = 2).

Supplementary Fig. 3
